# The need for translational research in respiratory medicine

**DOI:** 10.1186/2213-0802-1-9

**Published:** 2013-04-19

**Authors:** YC Gary Lee, Grant W Waterer

**Affiliations:** 1 School of Medicine & Pharmacology, University of Western Australia, Perth, Australia; 2 Centre for Asthma, Allergy & Respiratory Research, University of Western Australia, Perth, Australia; 3 Respiratory Department, Sir Charles Gairdner Hospital, Perth, Australia; 4 Respiratory Department, Royal Perth Hospital, Perth, Australia; 5 University Department of Med, Sir Charles Gairdner Hospital, 4/F, G Block, Perth, WA 6009 Australia

**Keywords:** Translational, Respiratory, Pneumonia, Pleural, Research, Empyema

## Abstract

Medical advances have failed to arrest the growing morbidity and mortality from lung diseases. COPD, lung cancer and pulmonary infections remain leading causes of death. More than any other time in human history, we need high quality, translatable, patient-focussed respiratory research that will improve clinical practice. Close teamwork of scientists and clinicians are essential. The results of these work need to be disseminated quickly and widely. The creation of an open access journal, such as Translational Respiratory Medicine, dedicated to translational respiratory research can help foster progress.

Lung diseases claim more lives now than they ever have. Chronic obstructive airways disease (COPD) will become the third leading cause of death according to predictions by the World Health Organization [1]. Lung carcinomas kill more people than any other cancers [2] and its increasing occurrence in non-smokers provokes further concerns. Medical advances over the past centuries have failed to eradicate lethal microbes such as tuberculosis [3], which was responsible for 1.4 million deaths in 2011 [4]. Pneumonia and pleural infection [5], once thought would extinct with advent of antibiotics, are also rising in incidences.

Advances in respiratory medicine are failing to meet the growing challenges from a diverse range of lung diseases [6]. More virulent and drug-resistant respiratory pathogens continue to emerge. Multi- (and even total-) drug resistant M. TB, penicillin-resistant pneumococci, methicillin-resistant *Staphylococcus aureus*, and carbapenem-resistant Enterobacteraciae are now commonplace. The massive global impact of SARS and H1N1 2009 influenza A have highlighted how vulnerable mankind remains in the ongoing war against our ever-evolving microbial enemies.

More than any other time in human history, we now need high quality, translatable, patient-focussed respiratory research - and fast! Resources for research are limited, and prioritization essential. Although ‘pure’ basic science is vital and major findings (e.g. discovery of the double helix) have profound implications, translational research to bring bench advances to the bedside is critical.

Success of translational research should be gauged by how (and how much) the result changes clinical practice. Meaningful translational research can only be achieved through close teamwork of scientists and clinicians at the frontline of clinical care.

The leap is massive from cell biology experiments performed under optimized laboratory conditions to successful outcomes in clinical trials which have to incorporate numerous inevitable (and uncontrollable) confounding factors inherent in human diseases. Animal models may be used to bridge the gap, but faithful models for human diseases are few and far between. All too often researchers overlook dissimilarities between their animal models and the human diseases under study, and such mistakes have been proven costly. When promising bench work fails to translate into clinical care, it sends years of hard work and millions of funding down the drain. The despair and frustration to the researchers are often beyond description.

Pneumonia research provides many such examples. Disease-modifying agents such as anti-tumor necrosis factor-alpha, granulocyte colony stimulating factor and tissue factor pathway inhibitor all performed splendidly in animal models but failed dismally in human clinical trials [7]. In the preclinical models used, pneumonia was induced acutely by intra-tracheal installation of large boluses of bacteria into animals. This is far removed from the slow onset of disease in humans which is often also modified by comorbidities of the host. It is no surprise that what works well in the mouse pneumonia model frequently does not work in man.

Another example is the mouse model of a single intra-tracheal instillation of bleomycin. For many years this model was used to examine disease pathogenesis and evaluate therapeutic targets for idiopathic pulmonary fibrosis (IPF), despite many differences in the anatomical distribution, disease chronicity and the level of acute lung inflammation between human IPF and bleomycin injured murine lungs. It is nowadays generally accepted that the bleomycin model is a better model for acute lung injury and not one of IPF [8].

These examples highlight the need for tight collaboration between bench and clinical researchers. Both must remain critical of existing models and be highly aware of the limitations of all *in vitro* and preclinical models employed.

## Successful examples of translational research

The path from the laboratory to the bedside is now far more complex than in past decades. The modern ethical framework for conducting research has created the necessary barriers to safeguard patient safety. Thankfully, no longer can clinicians simply take a drug under development and experiment it on patients, without evidence of its potential efficacy and safety data on its toxicology and teratogenicity.

The importance of full translational research in the workup of a therapeutic product is highlighted in the use of talc for pleurodesis [9, 10]. Millions of patients have received talc pleurodesis since the first report of talc poudrage in 1935 [11], even though talc has never been subjected to ‘proper’ laboratory workup and testing. Only half a century later was the observation made that talc could induce acute respiratory distress syndrome (ARDS). In subsequent studies, not only did researchers find impurities in many commercial talc preptions, they also discovered that talc particles were heterogenous in size within most products. Animal and human studies, including randomized trials, found that small talc particles are often systemically absorbed after their intrapleural delivery and induce lung inflammation and ARDS [12]. Talc-induced respiratory failure can affect up to 9% of patients, and caused death in 2.3% of the 482 patients undergoing talc pleurodesis in a multicenter study [13]. Clinical guidelines now recommend only the use of large particle size talc for pleurodesis [14].

On the other hand, there have been many significant successes in bringing laboratory discoveries to clinical care through proper translational research and regulatory channels. Conjugate pneumococcal vaccines, for example, have reduced the burden of disease in children and adults worldwide since their introduction [15]. Anti-human immunodeficiency virus agents have prolonged life expectancy in HIV patients by decades, and the discovery of EGFR mutations in lung cancer have led to new therapies for those harboring such mutations. Understanding the pathogenesis of cystic fibrosis (CF) has led to the first successful introduction of a modulator of CF transmembrane receptor, albeit only for patients with a G551D mutation [16].

## The role of the translational respiratory medicine journal

“*Research not published is as good as research never performed*”. A journal dedicated to the field of translational respiratory research can only foster progress. Open access journals allow wider dissemination of information. The creation of Translational Respiratory Medicine is therefore an exciting and right step towards the above stated goals.

“*Good beginning is half the success*” as the famous Chinese saying states. We congratulate Professors Bai, Matthay and Wang for their effort in establishing the TRM journal. The other half of the success now depends on the support from researchers around the world to rally and support this opportunity of developing a unique and prestigious forum for translational respiratory medicine.
